# Supplementary material for: Bias in product availability estimates from contraceptive outlet surveys: Evidence from the Consumer’s Market for Family Planning (CM4FP) study
Source: PLoS One. 2022 Aug 30;17(8):e0271896. doi: 10.1371/journal.pone.0271896 (PMC9426883; doi:10.1371/journal.pone.0271896)
Supplement: S2 File — A brief description of population estimates for pseudo-enumeration areas. (PDF) [file pone.0271896.s002.pdf]

S2 File. Pseudo-EA population estimation.

A brief description of population estimates for pseudo-enumeration areas.

Because of differences in study site scale and population density, different sizes of pseudo-EA likely correspond most closely to the true scale and population of census EAs that would be used for sampling of other outlet surveys, such as PMA. Pseudo-EA population figures were estimated by summing gridded population figures in six sets of EAs for each study site. Each set of EAs was a different size, scaled as a fraction of the size of the study site. The gridded population datasets are from www.worldpop.org [1, 2, 3]. The spatial resolution is 100m x 100m. Estimates are constrained to match 2020 United Nations population estimates at the country level and further constrained to only assign population to settled portions of the countryside. Each population grid cell that fell within the study site was assigned to a pseudo-EU using a point-in-polygon algorithm and then the populations were summed in each EA. Table 1 summarizes values for the study sites and six sets of EA sizes.

References

1. Bondarenko M., Kerr D., Sorichetta A., and Tatem, A.J. Census/projection-disaggregated gridded population datasets, adjusted to match the corresponding UNPD 2020 estimates, for 51 countries across sub-Saharan Africa using building footprints (Kenya). WorldPop, University of Southampton, UK. 2020 Sept. doi:10.5258/SOTON/WP00683.
2. Bondarenko M., Kerr D., Sorichetta A., and Tatem, A.J. Census/projection-disaggregated gridded population datasets, adjusted to match the corresponding UNPD 2020 estimates,

for 51 countries across sub-Saharan Africa using building footprints (Nigeria). WorldPop, University of Southampton, UK. 2020 Sept. doi:10.5258/SOTON/WP00683.

3. Bondarenko M., Kerr D., Sorichetta A., and Tatem, A.J. Census/projection-disaggregated gridded population datasets, adjusted to match the corresponding UNPD 2020 estimates, for 51 countries across sub-Saharan Africa using building footprints (Uganda). WorldPop, University of Southampton, UK. 2020 Sept. doi:10.5258/SOTON/WP00683.
